# Supplementary material for: Ultrastrong exciton-plasmon couplings in WS2 multilayers synthesized with a random multi-singular metasurface at room temperature
Source: Nat Commun. 2024 Apr 17;15:3295. doi: 10.1038/s41467-024-47610-z (PMC11024105; doi:10.1038/s41467-024-47610-z)
Supplement: Supplementary file 1 — Supplementary Information [file 41467_2024_47610_MOESM1_ESM.pdf]

Supplementary Materials for

Ultrastrong exciton-plasmon couplings in WS_2 multilayers synthesized with a random multi-singular metasurface at room temperature

Tingting Wu[1,#], Chongwu Wang[1,#], Guangwei Hu[1,#], Zhixun Wang[1], Jiaxin Zhao[2], Zhe Wang[1], Ksenia Chaykun[2], Lin Liu[1], Mengxiao Chen[3], Dong Li[4], Song Zhu[1], Qihua Xiong[5], Zexiang Shen[2], Huajian Gao[4], Francisco J. Garcia-Vidal[6,7,*], Lei Wei[1,*], Qi Jie Wang[1,2,*] and Yu Luo[8,*]

[1]School of Electrical and Electronic Engineering, Nanyang Technological University, Singapore, Singapore.
[2]School of Physical and Mathematical Sciences, Nanyang Technological University, Singapore, Singapore.
[3]Zhejiang Provincial Key Laboratory of Cardio-Cerebral Vascular Detection Technology and Medicinal Effectiveness Appraisal, Zhejiang University, Hangzhou, China
[4]School of Mechanical and Aerospace Engineering, Nanyang Technological University, Singapore, Singapore.
[5]State Key Laboratory of Low-Dimensional Quantum Physics and Department of Physics, Tsinghua University, Beijing, China.
[6]Departamento de Física Teorica de la Materia Condensada and Condensed Matter Physics Center (IFIMAC), Universidad Autónoma de Madrid, 28049 Madrid, Spain
[7]Institute of High Performance Computing, Agency for Science, Technology and Research (A*STAR), Connexis, 138632, Singapore
[8]National Key Laboratory of Microwave Photonics, Nanjing University of Aeronautics and Astronautics, Nanjing, China
[#]These authors contributed equally: Tingting Wu, Chongwu Wang, Guangwei Hu.
[*]emails: yu.luo@nuaa.edu.cn; qjwang@ntu.edu.sg; wei.lei@ntu.edu.sg; fj.garcia@uam.es

Supplementary Note 1 Dense packing of sub-nanometer plasmonic gaps by cold-etching technique

Two main paths are identified to achieve sub-nanometer gaps. The first is to apply a stress to the PEI substrate that exceeds the critical value (to ensure the onset of the fracture across the metal strip/domain at width L_c) while approaching saturation (where the fracture across the metal strip/domain stops as the stress transferred from the PEI substrate is insufficient to further break the existing fragment strip/domain at width $L_s < L_c$) by selecting appropriate mechanical loadings[1]. The critical stress applied to the PEI substrate to break the fragment strip/domain with thickness of h, width of L (perpendicular to the loading direction), and fracture strength of σ^* (which is a constant or varies slightly with the thickness of the metal film) is $\sigma_c = 2h\sigma^*/L$, showing that a narrower strip/domain requires a greater applied stress to break under the condition of fixed thickness. Thus, an appropriate mechanical loading can generate a new crack near the centre of a preformed strip/domain, while the stress transferred from the PEI substrate has an opportunity to saturate somewhere in the narrow region of the strip/domain, and the corresponding crack does not extend too far and stops before the narrow region completely breaks into two parts, resulting in a sub-nanometer fracture gap in the preformed strip/domain (Fig. S2). It should be noted that the strip/domain is not uniform in width (wider at some random locations and narrower at others due to the intergranular fracture), and the tensile stress is maximum at the centre of the strip/domain under mechanical loadings, which are the reasons why the newly generated crack starts from the centre of the wide part of the strip/domain. The second path is to use biaxial intergranular fractures to create sub-nanometer distances between adjacent fragment domains, where the deflected crack edges of one fragment domain are dragged close to the crack edges of the adjacent fragment at sub-nanometer spacings (Fig. S3).

In order to elucidate the mechanism of the packing of sub-nanometer plasmonic gaps in the cold-etched plasmonic metasurfaces, the fracture morphology and the stress-strain relationship were characterized in detail from a geometrical point of view. The strain-stress behaviour of the gold-amorphous PEI polymers with different gold film thicknesses is shown in Fig. S4a. In the *x*-directional stretching (Fig. S4a), the necking process is located in the middle strain range with constant stress, and the strain is localized in this region. The advantage of necking in plastic polymers is that, compared to elastic polymers (without the necking process), the fragmenting of metal films can be well controlled with roughly homogeneous fracture strips[2]. The zigzag crack deflection angles (from the mechanical loading direction) were between 55° and 65° (Fig. S5), analogous to the crack deflection angles for mixed local thinning and intergranular fracture[3]. The strain-stress curve during the *y*-directional (2nd) stretching (Fig. S4b) behaves as in elastic polymers, where the stress in the polymer increases with increasing strain without necking because the polymer chains are already well aligned during necking, and the plastic polymer film (plastic refers to the irreversible deformation of the polymer when the tensile force is released) stretches homogeneously over the entire length.

The packing density of the sub-nanometer plasmonic gaps in the gold film under tension is expected to be determined by the mechanical properties of the plastic polymer used for cold-etching (Fig. S4), the thickness of the deposited gold film, and the fracture strain of the gold film/polymer sample. The SEM images of the fracture morphology clearly show the packing of sub-nanometer plasmonic gaps.

Supplementary Note 2 Geometrical and optical isotropy

We have investigated the isotropic/anisotropic properties of the WS_2 monolayer coupled multi-singular metasurface system from geometrical and optical perspectives, by exploring the fracture morphology and the corresponding polarization dependence of the PL emission and the coupling strength (between the WS_2 excitons and plasmons). Geometrical isotropy/anisotropy refers to the average fragment domain size in different directions, which depends on the elongation extents of the biaxial stretching. Anisotropy here means that the average fragment domain size is different in one direction than in the other. When the elongation is 0%, the gold film breaks into approximately uniform strips with the greatest geometrical anisotropy (Fig. S6d). The geometrical anisotropy decreases and becomes isotropy at about 80% of elongation (Fig. S6a-c) as it increases from 0% to 80%, and the opposite geometrical anisotropy appears above 80% (Fig. S6e,f). It should be noted that the cracks under mechanical loading are along random grain boundaries of the gold film, so only two perpendicular mechanical loadings can produce isotropic cold-etched metallic metasurfaces. The polarization-resolved coupling strength (Fig. S11) confirms the isotropic/anisotropic optical properties. The coupling strength between WS_2 excitons and plasmons was fitted by the full Hopfield Hamiltonian. The isotropic and anisotropic behaviour of the coupling strength is in good agreement with the geometrical perspective (Fig. S6), i.e., for metasurfaces with different gold film thicknesses, the coupling strength is isotropic at 80% *y*-directional elongation, for the 20 nm thick metasurface case, the maximum anisotropy occurs at 0% *y*-directional elongation, and the opposite anisotropy occurs at 100% *y*-directional elongation. Thus, there is a one-to-one correspondence between optical isotropy/anisotropy and geometrical isotropy/anisotropy. Among the geometrically isotropic metasurfaces, the metasurface with a 20 nm thick gold film has the best coupling. This best performance is provided by the smallest plasmonic mode volume of the 20 nm thick gold isotropic metasurface due to its dense sub-nanometer plasmonic gaps and a certain thickness of the gold film.

Our results show that cold-etching is effective in modifying the in-plane fracture behaviour (i.e., in-plane crack extension) of metal thin films by controlling the packing of the sub-nanometer plasmonic gaps of the plasmonic metasurfaces, and the corresponding isotropic/anisotropic properties. The cold-etched metasurface has a unique ease of manufacture and a high degree of tunability.

Supplementary Note 3 WS_2 coupled antennas fabrication

The bowtie and dimer antennas were fabricated using the electron beam lithography (EBL) lift-off process. EBL patterning was achieved by a series of line exposures at 30 kV energy and 15 pA beam current on 80 nm-thick PMMA e-beam resist, followed by cold development in a mixture of MIBK and isopropyl alcohol. A 20 nm-thick gold film was then deposited on the sample using an e-beam evaporator, and the lift-off pattern transfer was performed in acetone for 10 min. WS_2 monolayers were transferred to the antennas using a dry transfer method.

Supplementary Note 4 Theoretical fitting

In the theoretical fitting process, we first extract the characteristic polariton energies (i.e., lower (ω_-) and upper (ω_+) polaritons) from the scattering peaks in the spectra in Fig. 2a,b. We then

calculate the coupling strength ($g = 165.9$ meV at 0 strain and $g = 240.4$ meV at -2% strain) by fitting the dark-field spectra in Fig. 2a,b to a coupled oscillator model[4] where each scattering spectrum is calculated by $S(\omega) \propto \omega^4 \left| \frac{\omega_{ex}^2 - \omega^2 - i\gamma_{ex}\omega}{(\omega_{pl}^2 - \omega^2 + i\gamma_{pl}\omega)(\omega_{ex}^2 - \omega^2 + i\gamma_{ex}\omega) - \omega^2 g^2} \right|^2$. Here, only g is the unknown parameter, and ω_{ex} (known value) and ω_{pl} (calculated from the extracted ω_- and ω_+ as $\omega_{pl} = \omega_+\omega_-/\omega_{ex}$) are the energies of the WS_2 excitons and the plasmonic mode, respectively, and $\gamma_{pl} = 380$ meV and $\gamma_{ex} = 45$ meV are the damping losses of plasmonic resonance and exciton emission, respectively. Third, we fit the polariton energies (ω_+ and ω_- extracted from the scattering peaks) as eigenvalues of the full Hopfield Hamiltonian[5,6] which yields $(\omega^2 - \omega_{ex}^2)(\omega^2 - \omega_{pl}^2) - 4g^2\omega^2 = 0$.

Note that, we do not need to use Au thickness parameters in the theoretical fitting process. In Fig. 2c of the original manuscript, the *x*-axis is the Au thickness showing that different ω_{pl} are introduced by different Au thicknesses. Different Au thicknesses are used to obtain different plasmonic resonances ω_{pl} and thus different detunings ($= \omega_{pl} - \omega_{ex}$, which is the difference between the plasmonic resonance and the exciton energy) to obtain anti-crossing. In fact, different detunings can be obtained in conventional plasmonic metasurfaces by using various techniques, for example, different detunings can be obtained in the well-known gold bowtie nanoantennas by introducing different metal film thicknesses (as in our case), different plasmonic gaps, or different bowtie side lengths, etc.

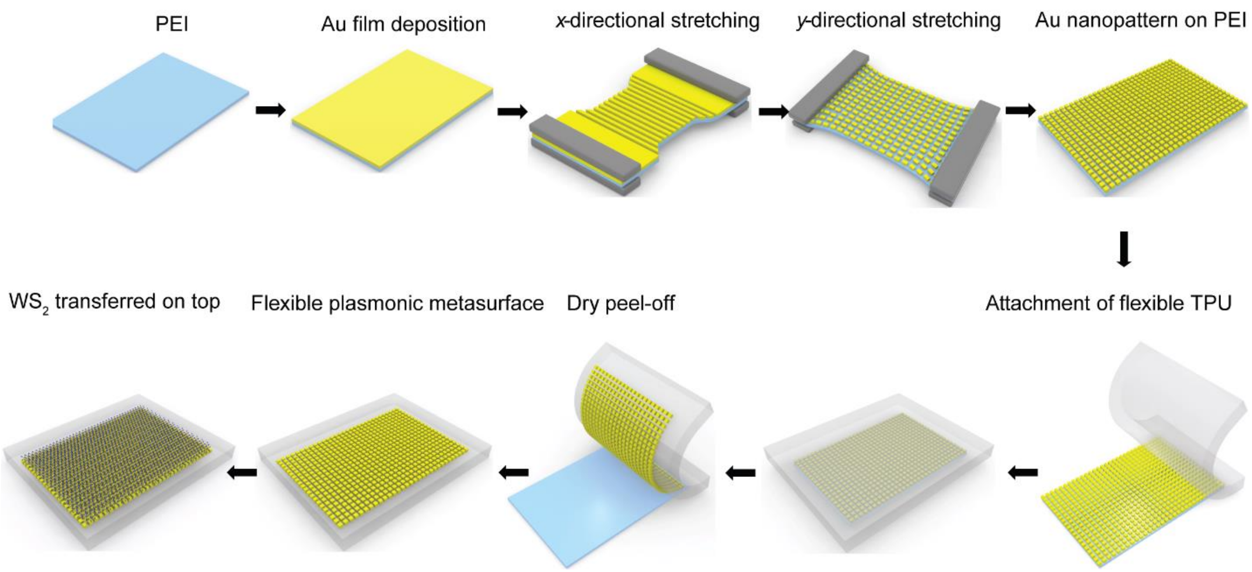

Supplementary Figure 1 Schematic illustration of the fabrication process of the WS_2 monolayer coupled multi-singular plasmonic metasurface. A thin gold film was deposited on a PEI polymer substrate (125 μm thick, smooth on both sides) using an electron beam evaporator at a deposition rate of 2 $Ås^{-1}$. During the evaporation, the temperature of the vacuum chamber was kept below 60 °C throughout the whole process, to prevent the thermal expansion or deformation of the PEI film due to built-in stress, which would cause wrinkles or defects in the gold film. For cold-etching, the first stretch was conducted by stretching the gold/PEI film in the *x*-direction. The second 2D stretch was conducted by re-stretching the as-fabricated film in the *y*-direction. After the cold-etching, the resulting gold nanopattern was transferred to a flexible TPU polymer substrate (2 mm thick) using a dry peel-off method. WS_2 monolayers were mechanically exfoliated from the purchased commercial bulk WS_2 crystals using a PDMS tape and transferred to the gold nanopatterns (on TPU) using a dry transfer method. This cold-etching technique is easy to implement, time-saving, low-cost, and large-scale self-assembly. The above sample preparation process demonstrates that cold-etching technology is easy to implement, time-saving, low-cost, and large-scale self-assembly. PEI: poly(ethyleneimine) polymer; Au: gold; TPU: thermoplastic polyurethane polymer; PDMS: poly(dimethylsiloxane) polymer.

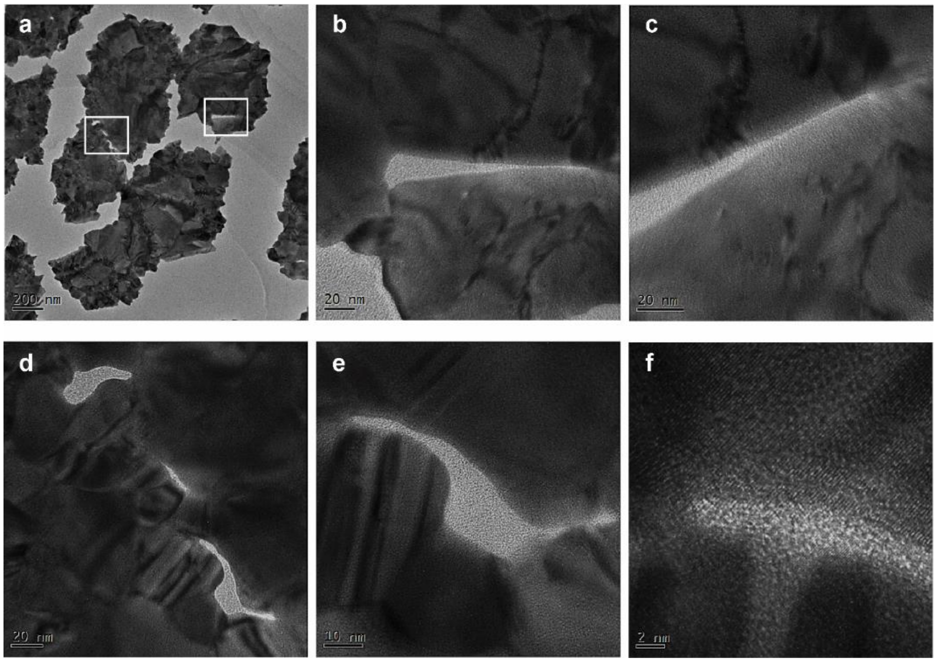

Supplementary Figure 2 Transmission electron microscopy (TEM) image of the random multi-singular metasurface. a, TEM images of the 20 nm thick gold multi-singular metasurface showing the densely packed sub-nanometer plasmonic gaps from the first path. **b-f**, Enlarged view of the section within the white box in **a**, showing the sub-nanometer gap more clearly. A new crack is created in the preformed strip/domain, extends and stops before the strip/domain completely breaks into two, resulting in a sub-nanometer fracture gap in the preformed domain.

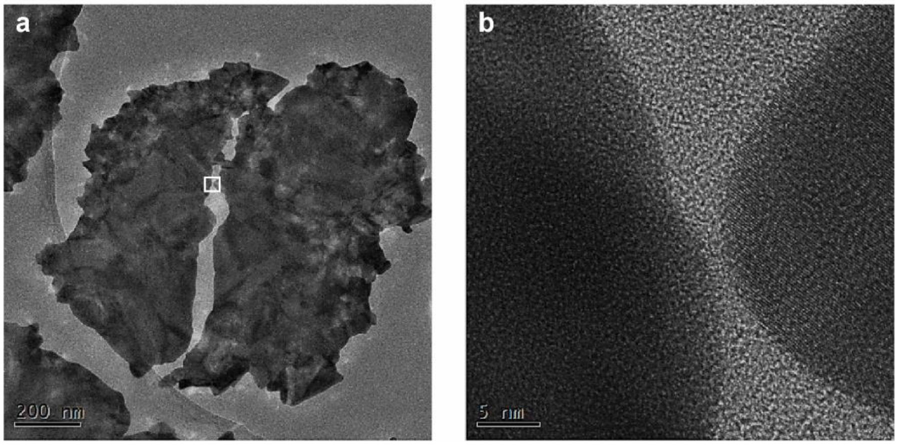

Supplementary Figure 3 TEM image of the random multi-singular metasurface. a, TEM image of the 20 nm thick gold multi-singular metasurface showing the densely packed sub-nanometer plasmonic gaps from the second path. **b**, Enlarged view of the section within the white box in **a**. Biaxial intergranular fractures are used to create sub-nanometer distances between adjacent fragment domains, where the deflected fracture edges of one fragment domain is dragged close to the fracture edges of the adjacent fragment domains at sub-nanometer spacing.

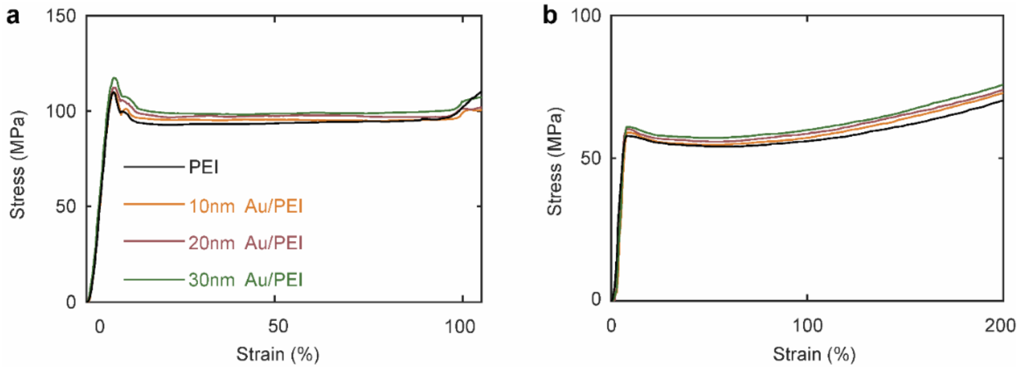

Supplementary Figure 4 Stress-strain curves. Stress-strain curves for the gold film (20 nm thick)/PEI (125 μm thick) substrate in **a**, *x*-directional and **b**, *y*-directional stretching processes, respectively.

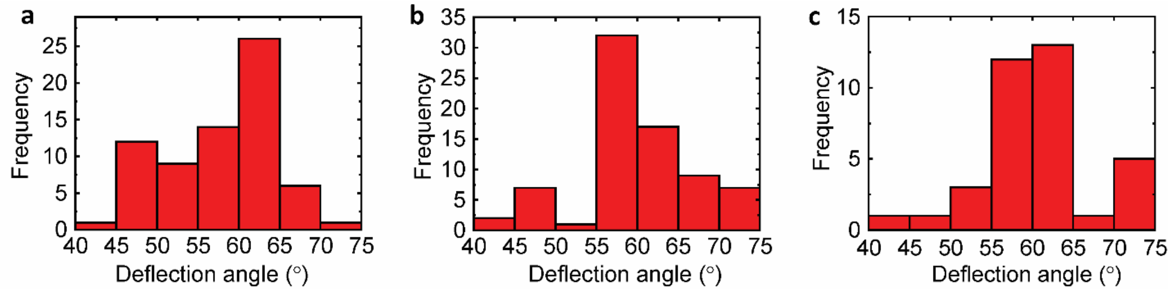

Supplementary Figure 5 Statistical analysis of the zigzag crack deflection angles. a-c, Statistical analysis of the zigzag crack deflection angles (from the loading direction) in the x-directionally stretched plasmonic metasurface. The gold film thicknesses are **a**, 10 nm, **b**, 20 nm, and **c**, 30 nm, respectively. The zigzag crack deflection angles are between 55° and 65°.

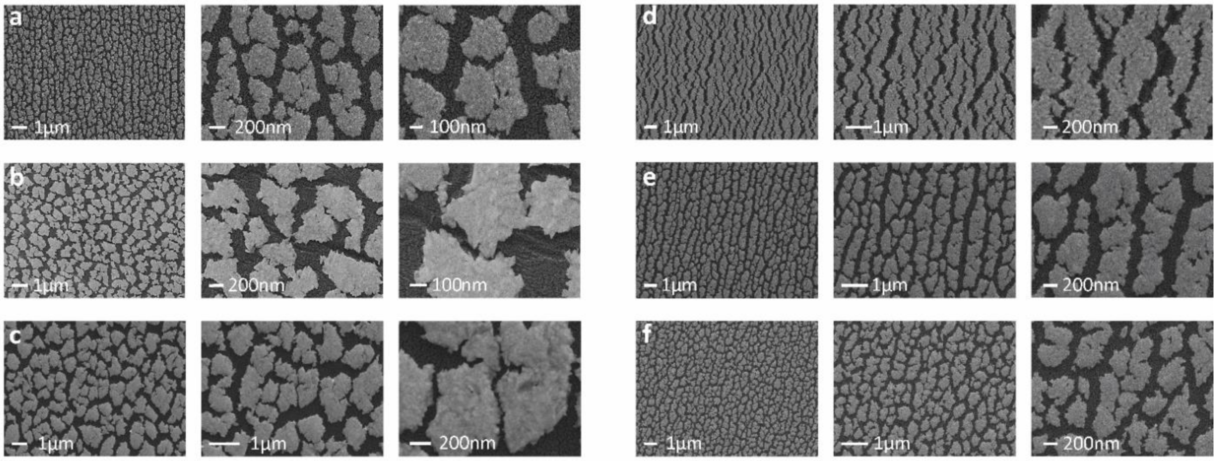

Supplementary Figure 6 SEM images of the metasurface with different y-directional elongation. a-c, SEM images of cold-etched isotropic metasurfaces with metal layer of (**a**) 10 nm, (**b**) 20 nm, and (**c**) 30 nm, respectively. Isotropic refers to the case where the y-directional stretching has the same elongation as the x-directional stretching (~80%). **d-f**, SEM images of the anisotropic metasurface with a gold layer thickness of 20 nm with y-directional elongation at 0% (**d)**, 56% (**e**), and 100% (**f**), respectively. Anisotropy here means that the average fragment domain size is different in one direction than in the other. Note that, despite of the random distribution and orientation of the singularities, the multi-singular metasurface maintains a 'global uniformity', which refers to the uniform distribution of singularities within the laser beam area or across the entire patterned gold film, as observed in SEM images. The global uniformity can be precisely controlled by the y-directional elongation.

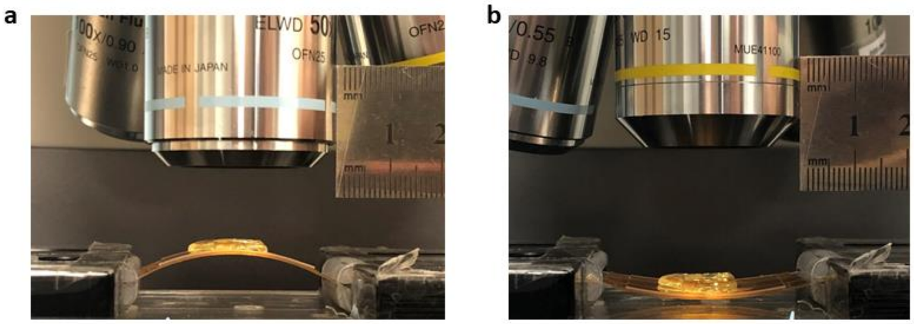

Supplementary Figure 7 Experimental setup to apply uniaxial strain by bending the flexible substrate. **a**, The positive strain (upward bending) is tensile on the ‘outside’ surface. **b**, The negative strain (downward bending) is compressive on the ‘inside’ surface.

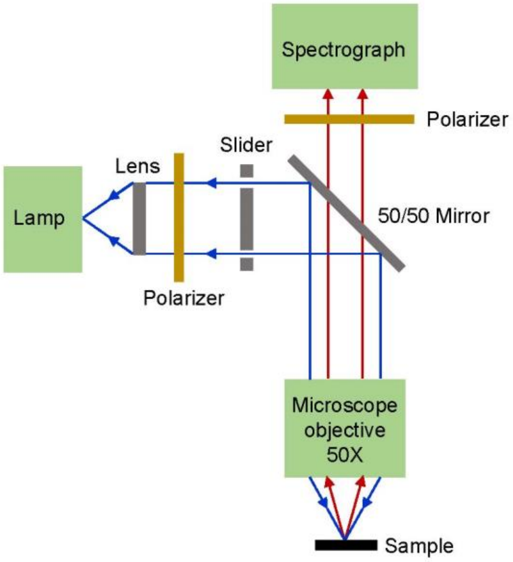

Supplementary Figure 8 Schematic of the dark-field scattering measurement setup. A hyperspectral imaging system with a broadband halogen lamp is used as the light source. A 50× objective was used, and the incident light power was ~20 μW with a laser spot size of ~5 μm.

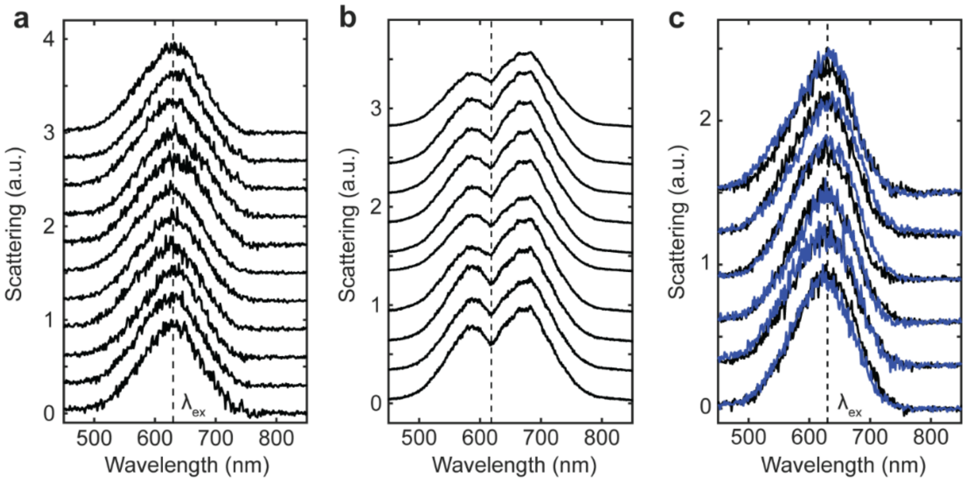

Supplementary Figure 9 Dark-field scattering spectra. Dark-field scattering spectra measured at 10 different random positions of a 20 nm thick Au metasurface **a**, without and **b**, with monolayer WS_2 on top. The relative standard deviation values at the resonance wavelength/bandwidth of the bare plasmons and the Rabi splitting are 6.2%/8.2% and 7.5%, respectively, indicating the stability and repeatability of our random multi-singular metasurface. **c**, Dark-field scattering spectra at 6 different random positions of a 20 nm thick Au metasurface. Black lines correspond to the signal of a newly prepared sample, blue lines correspond to the signal repeated 12 months after the samples were fabricated at the same sample positions. No significant difference in the scattering spectra when the measurements were repeated 12 months after the samples were fabricated, further demonstrating the stability and robustness of the plasmonic metasurfaces.

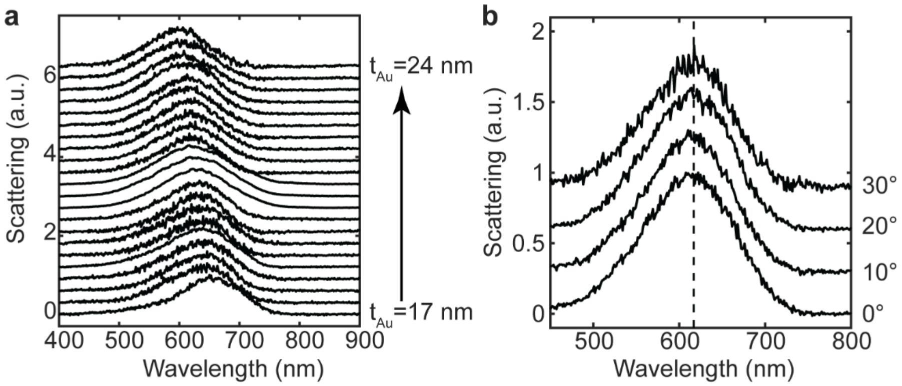

Supplementary Figure 10 Dark-field scattering spectra. Dark-field scattering spectra of the metasurface **a**, with different gold film thicknesses (from bottom to top, the gold film thickness (t_{Au}) increases from 17 nm to 24 nm) and **b**, at different angles of incidence (0° to 30° with Au thickness is 22 nm).

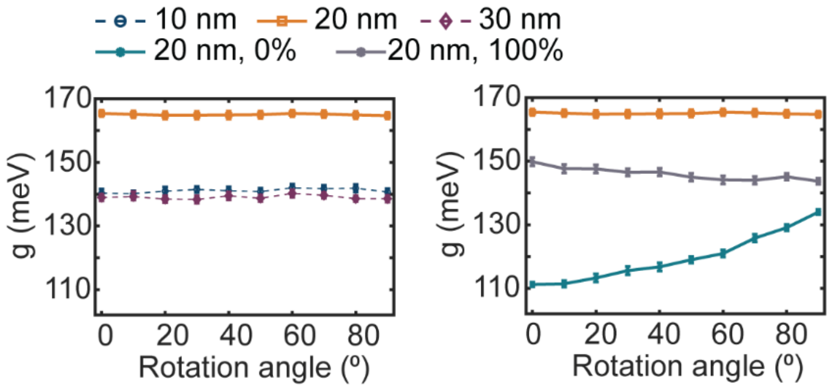

Supplementary Figure 11 Coupling strength (right panel) for different plasmonic systems. The percentage indicates the y-directional elongation extent. Curves in the figure labelled only with the thickness value but not with elongation extent represent isotropic samples.

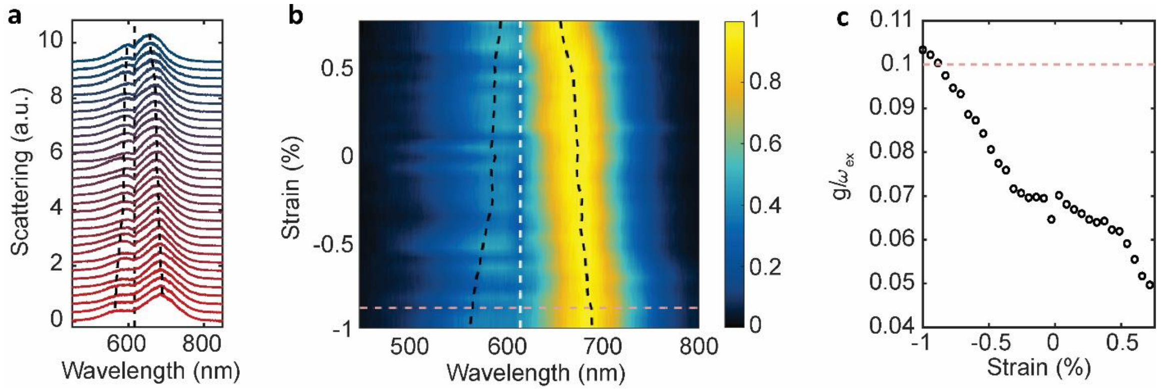

Supplementary Figure 12 Active ultrastrong coupling of different plasmonic metasurfaces from Fig. 3 of the main paper. a, Representative dark field scattering spectra at different uniaxial strains. The strain value gradually increases from red (-1%) to blue (0.75%) and the interval is ~0.06%. **b**, Dark field scattering spectra as a function of excitation wavelength and strain. **c**, Normalized coupling strength as a function of the uniaxial strain. The horizontal dashed line marks the onset of ultrastrong coupling.

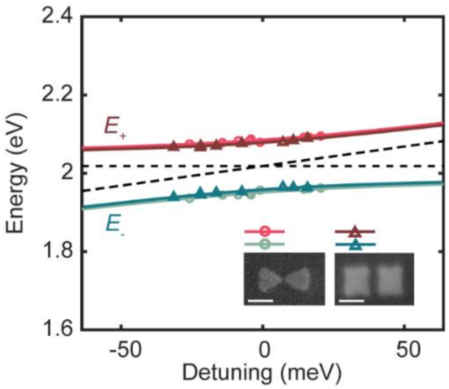

Supplementary Figure 13 Strong exciton-plasmon coupling in a WS_2 monolayer coupled to bowtie and dimer antennas. The upper and lower polariton branches of the WS_2 coupled bowtie (circles) and dimer (triangles) antenna systems are fitted (solid lines) with a coupling strength of 80.9 meV and 86 meV, respectively, in the Tavis-Cummings Hamiltonian, respectively. The plasmonic mode frequencies for the two traditional plasmonic systems are almost overlap (oblique dashed lines). The scale bar is 100 nm.

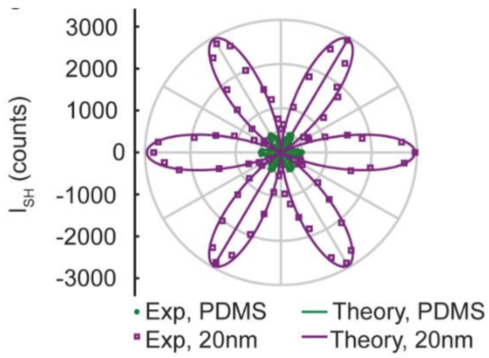

Supplementary Figure 14 Polarized polariton-enhanced SHG from the WS_2 monolayer. A typical 6-fold symmetric petal SHG pattern is well fitted by $I_{SH} = C\cos^2 3\theta$ showing the isotropic property of the multi-singular plasmonic metasurface, where C is the maximum SHG intensity, and θ is the rotation angle of the sample. The intensity of the SHG emission from the WS_2 on PDMS is amplified by a factor of three for a clear view. The polariton-enhanced SHG from the WS_2 monolayer is around 15 times stronger than that on PDMS. The polarization-independent polariton-enhanced SHG is promising because it eliminates the need to align the crystal orientation of the WS_2 for effective polariton-enhanced nonlinearity, in contrast to the polarization-dependent SHG in the WS_2 coupled bowtie antenna system where maximum enhancement occurs only when the pump field is polarized along the longitudinal symmetry axis.

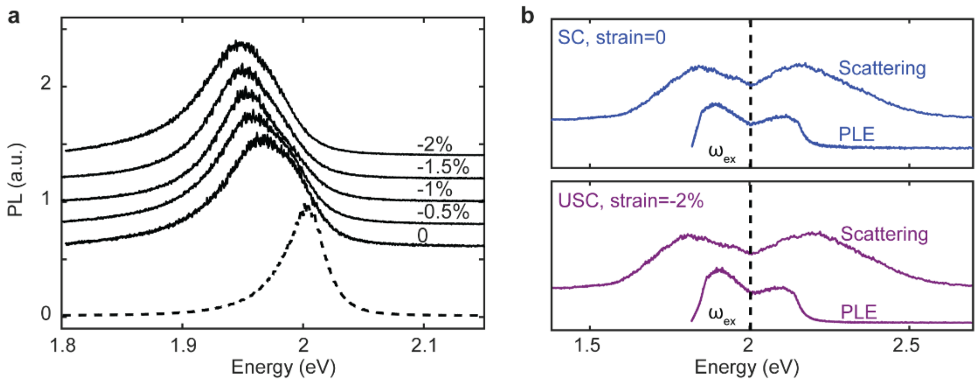

Supplementary Figure 15 PL and PLE. a, Normalized photoluminescence (PL) intensity under different strains. We find no splitting in the PL emissions of the coupled plasmonic system; instead, we observe a noticeable red shift of the PL emission peak position as in other previous work. Our inability to observe Rabi splitting in the PL measurements is due to the large laser spot size (diameter~4 μm) and the resulting high fraction of background PL contribution (emission from the uncoupled exciton, dashed line). **b**, Normalized PLE ($\mathrm{PLE} = (\mathrm{PL}_{\mathrm{WS_2\ on\ metasurface}} - \mathrm{PL}_{\mathrm{WS_2\ on\ polymer}})/\mathrm{PL}_{\mathrm{WS_2\ on\ polymer}})$ compared to scattering in the SC (upper) and USC (lower) regime, respectively. The splitting in the PLE is narrower than that in the scattering cross sections, as has been verified. We attribute this to the absorption and scattering paths associated with the PLE and dark-field scattering processes, respectively. Note that the upper polariton branch is not shown in PL spectra. This is because in the strongly coupled systems, part of the detected PL ($\mathrm{PL}_{\mathrm{WS_2\ on\ metasurface}}$) is emitted by the radiation of plasmon-exciton hybrid modes, which can modulate the PL spectra, and part of the detected PL signal is attributed by the background PL from the uncoupled excitons ($\mathrm{PL}_{\mathrm{WS_2\ on\ polymer}}$). This makes it difficult to extract the strong coupling information from the PL spectra. Separating the PL emission due to the radiation of plasmon-exciton hybrid modes from that due to uncoupled excitons (i.e., $\mathrm{PLE} = (\mathrm{PL}_{\mathrm{WS_2\ on\ metasurface}} - \mathrm{PL}_{\mathrm{WS_2\ on\ polymer}})/\mathrm{PL}_{\mathrm{WS_2\ on\ polymer}}$) can help to reveal the strong coupling induced spectral features of PL.

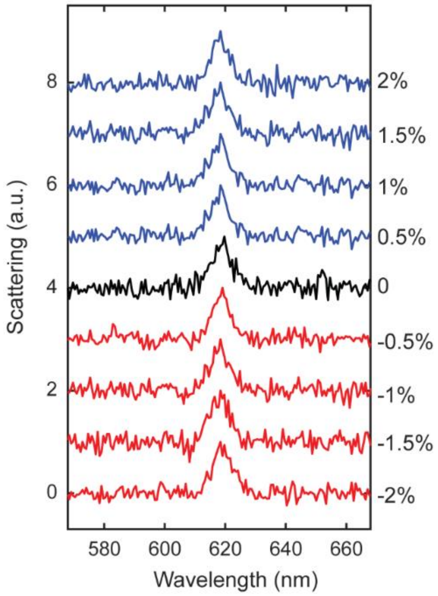

Supplementary Figure 16 Effect of strain on WS_2 exciton energy. Dark field scattering spectra of WS_2 monolayer on PDMS substrate under different strains.

Supplementary Table 1. Emitter thickness and corresponding normalized coupling strength (g/ω_{ex}), coupling strength (g), and exciton energy (ω_{ex}) in this work (purple), TMDs (red), organic molecules (blue), and inorganic quantum dots (QD, green) with a thickness less than 20 nm.

System	Thickness (nm)	g/ω_{ex}	g(meV)	ω_{ex}(eV)
This work	0.618	0.12	240.4	2.0189
	1.854	0.147	295.5	2.01
	2.472	0.164	328.5	2.003
1L WSe_2/PC	0.649	0.006	9.5	1.6[7]
1L $MoSe_2$/DBR	0.646	0.014	23	1.66[8]
1L $MoSe_2$/DBR	0.646	0.005	7.6	1.66[9]
1L WS_2/DBR	0.618	0.01	20.8	2[10]
1L WS_2/DBR	0.618	0.01	20.3	2.078[11]
	0.618	0.01	19.8	2.072
	0.618	0.009	18.8	2.05
	0.618	0.009	18.3	2.042
1L WS_2/DBR	0.618	0.008	16.5	2[12]
1L MoS_2/DBR	0.615	0.015	28	1.855[13]
1L WS_2/Ag microcavity	0.618	0.025	50	1.98[14]
1L WS_2/Ag Prism	0.618	0.023	48	2.05[15]
1L MoS_2/Ag NP on Au film	0.615	0.051	95	1.864[16]
1L WSe_2/Ag Nanorod	0.649	0.015	24.75	1.659[17]
1L WS_2/Au Nanorod	0.618	0.027	53	1.950[18]
1L MoS_2/Ag disk array	0.615	0.033	63	1.937[19]
1L WS_2/Au metasurface	0.618	0.012	24	2.01[20]
1L WS_2/Au Prism	0.618	0.041	82	1.99[21]
1L WS_2/Au nanoantenna	0.618	0.035	69	2[22]
1L WS_2/Au nanoantenna	0.618	0.032	64	2.02[23]
1L WS_2/Ag particle	0.618	0.032	65	2.03[24]
	3.708	0.036	73.82	
1L WS_2/Chirped Ag nanogroove array	0.618	0.028	56.82	2.028[25]
1L,2L,4L,16L WS_2/Ag nanorod array	0.618	0.012	24.39	2.023[26]
	1.236	0.014	29.02	
	2.472	0.019	39.00	
	9.888	0.025	50.03	
1L WS_2/Au nanorods	0.618	0.015	28.64	1.96[27]
1L WSe_2/Au nanorods	0.649	0.03	49.78	1.67[28]
1L WS_2/Au@Ag nanocuboid	0.618	0.024	48.98	2.021[29]
1L, 2L, 3L, 4L, 8L WSe_2/Au bi-pyramids	0.649	0.027	44.71	1.67[30]
	1.298	0.027	45.84	
	1.947	0.03	49.86	
	2.596	0.031	52.07	
	5.491	0.031	51.85	
1L MoS_2/Au bi-pyramids	0.615	0.022	42.3	1.88[31]
1L, 7L WS_2/Au nanodisks	0.618	0.033	64.62	1.963[32]
	4.326	0.047	93	
1L WS_2/Ag nanoprisms	0.618	0.037	73.53	2.012[33]
12L WSe_2/Au NPs on Au film	7.788	0.044	71.36	1.63[34]
1L WSe_2/Ag nanocubes on Ag film	0.649	0.017	28.08	1.66[35]

1L WS_2/Ag nanocubes on Ag film	0.618	0.041	84.04	2.02[36]
1L MoS_2/Au nanospheres on Au film	0.615	0.035	65.11	1.865[37]
1L WS_2/Ga NPs on Au film	0.618	0.041	83.16	2.02[38]
TDBC J-aggregates/Ag nanorods	2	0.025	52.6	2.11[39]
TDBC J-aggregates/Ag nanoprisms	2	0.067	142.21	2.11[40]
TDBC J-aggregates/Ag nanoprisms	3	0.095	200.39	2.11[41]
PIC J-aggregates/Au@Ag cuboid nanorods	1	0.053	113.39	2.16[42]
TDBC J-aggregates/Au@Ag nanorings	2	0.053	111.3	2.116[43]
DPDC J-aggregates in PVA/Au nanodisk dimers	15	0.078	140.09	1.79[44]
Cyanine dye J-aggregates/Au NP dimers	5	0.043	91.55	2.14[45]
J-aggregates/Au nanocubes on Au film	3	0.048	91.73	1.91[46]
Atto 532 molecules/Ag nanocubes on Ag film	9	0.032	84.75	2.66[47]
CdSe/ZnS QDs/Ag nanobowties	8	0.06	108.66	1.8[48]
CdSe/ZnS QDs/Ag nanobowties	8	0.067	119.95	1.8[49]
CdSe/CdS QDs/Au NP dimer	8	0.09	188.52	2.09[50]
CdSe/CdS QDs/Au NPs on Ag film	6	0.06	115.97	1.92[4]
CdSe/ZnS QDs/Au tip above Au substrate	5	0.046	86.09	1.87[51]
Carbon QDs/Au NPs on Au film	0.34	0.039	71.59	1.82[52]

References

1. Yanaka, M., Tsukahara, Y., Nakaso, N. & Takeda, N. Cracking phenomena of brittle films in nanostructure composites analysed by a modified shear lag model with residual strain. *J. Mat. Sci.* **33**, 2111-2119 (1998).
2. Li, T. & Suo, Z. Ductility of thin metal films on polymer substrates modulated by interfacial adhesion. *Int. J. Solids Struct*. **44**, 1696-1705 (2007).
3. Hutchinson, J. W. & Suo, Z. Mixed mode cracking in layered materials. *Adv. Appl. Mech.* **29**, 63-191 (1991).
4. Leng, H., Szychowski, B., Daniel, M.-C. & Pelton, M. J. Strong coupling and induced transparency at room temperature with single quantum dots and gap plasmons. *Nat. Commun*. **9**, 4012 (2018).
5. Ciuti, C. & Carusotto, I. Input-output theory of cavities in the ultrastrong coupling regime: The case of time-independent cavity parameters. *Phy. Rev. A* **74**, 033811 (2006).
6. Hopfield, J. Theory of the contribution of excitons to the complex dielectric constant of crystals. *Phy. Rev.* **112**, 1555 (1958).
7. Zhang, L., Gogna, R., Burg, W., Tutuc, E. & Deng, H. Photonic-crystal exciton-polaritons in monolayer semiconductors. *Nat. Commun.* **9**, 1-8 (2018).
8. Lundt, N. et al. Optical valley Hall effect for highly valley-coherent exciton-polaritons in an atomically thin semiconductor. *Nat. Nanotechnol.* **14**, 770-775 (2019).
9. Dufferwiel, S. et al. Valley-addressable polaritons in atomically thin semiconductors. *Nat. Photon.* **11**, 497-501 (2017).
10. Liu, X. et al. Nonlinear optics at excited states of exciton polaritons in two-dimensional atomic crystals. *Nano Lett.* **20**, 1676-1685 (2020).
11. Liu, X. et al. Control of coherently coupled exciton polaritons in monolayer tungsten disulphide. *Phys. Rev. Lett.* **119**, 027403 (2017).
12. Gu, J., Chakraborty, B., Khatoniar, M. & Menon, V. M. A room-temperature polariton light-emitting diode based on monolayer WS_2. *Nat. Nanotechnol.* **14**, 1024-1028 (2019).
13. Chen, Y. J., Cain, J. D., Stanev, T. K., Dravid, V. P. & Stern, N. P. Valley-polarized exciton–polaritons in a monolayer semiconductor. *Nat. Photon.* **11**, 431-435 (2017).
14. Sun, Z. et al. Optical control of room-temperature valley polaritons. *Nature Photonics* **11**, 491-496 (2017).
15. Munkhbat, B. et al. Electrical control of hybrid monolayer tungsten disulfide–plasmonic nanoantenna light–matter states at cryogenic and room temperatures. *ACS Nano* **14**, 1196-1206 (2020).
16. Hou, S. et al. Manipulating coherent light–matter interaction: continuous transition between strong coupling and weak coupling in MoS_2 monolayer coupled with plasmonic nanocavities. *Adv. Opt. Mater.* **7**, 1900857 (2019).
17. Zheng, D. et al. Manipulating coherent plasmon–exciton interaction in a single silver nanorod on monolayer WSe_2. *Nano Lett.* **17**, 3809-3814 (2017).
18. Wen, J. et al. Room-temperature strong light–matter interaction with active control in single plasmonic nanorod coupled with two-dimensional atomic crystals. *Nano Lett.* **17**, 4689-4697 (2017).
19. Lee, B. et al. Electrical tuning of exciton–plasmon polariton coupling in monolayer MoS_2 integrated with plasmonic nanoantenna lattice. *Nano Lett.* **17**, 4541-4547 (2017).
20. Chervy, T. et al. Room temperature chiral coupling of valley excitons with spin-momentum locked surface plasmons. *ACS Photon.* **5**, 1281-1287 (2018).
21. Qin, J. et al. Revealing strong plasmon-exciton coupling between nanogap resonators and two-dimensional semiconductors at ambient conditions. *Phys. Rev. Lett.* **124**, 063902 (2020).
22. Liu, L. et al. Strong plasmon–exciton interactions on nanoantenna array–monolayer WS_2 hybrid system. *Adv. Opt. Mater.* **8**, 1901002 (2020).
23. Liu, L. et al. Plasmon-induced thermal tuning of few-exciton strong coupling in 2D atomic crystals. *Optica* **8**, 1416-1423 (2021).
24. Yankovich, A. B. et al. Visualizing spatial variations of plasmon–exciton polaritons at the nanoscale using electron microscopy. *Nano Lett*. **19**, 8171-8181 (2019).
25. Sang, Y. et al. Tuning of two-dimensional plasmon–exciton coupling in full parameter space: a polaritonic non-Hermitian system. *Nano Lett*. **21**, 2596-2602 (2021).
26. Wang, S. et al. Limits to strong coupling of excitons in multilayer WS_2 with collective plasmonic resonances. *ACS Photon*. **6**, 286-293 (2019).
27. Jiang, Y. et al. Resonance coupling in an individual gold nanorod–monolayer WS_2 heterostructure: photoluminescence enhancement with spectral broadening. *ACS Nano* **14**, 13841-13851 (2020).

28. Wen, J. et al. Room-temperature strong coupling between dipolar plasmon resonance in single gold nanorod and two-dimensional excitons in monolayer WSe_2. *Chinese Phys. B* **27**, 096101 (2018).
29. Wang, S. et al. Coherent coupling of WS_2 monolayers with metallic photonic nanostructures at room temperature. *Nano Lett.* **16**, 4368-4374 (2016).
30. Stuhrenberg, M. *et al.* Strong light–matter coupling between plasmons in individual gold bi-pyramids and excitons in mono-and multilayer WSe_2. *Nano Lett.* **18**, 5938-5945 (2018).
31. Lawless, J. et al. Influence of gold nano-bipyramid dimensions on strong coupling with excitons of monolayer MoS_2. *ACS Appl. Mater. Interfaces* **12**, 46406-46415 (2020).
32. Geisler, M. et al. Single-crystalline gold nanodisks on WS_2 mono-and multilayers for strong coupling at room temperature. *ACS Photon*. **6**, 994-1001 (2019).
33. Cuadra, J. et al. Observation of tunable charged exciton polaritons in hybrid monolayer WS_2-plasmonic nanoantenna system. *Nano Let*t. **18**, 1777-1785 (2018).
34. Kleemann, M. E. et al. Strong-coupling of WSe_2 in ultra-compact plasmonic nanocavities at room temperature. *Nat. Commun.* **8**, 1296 (2017).
35. Sun, J. et al. Light-emitting plexciton: exploiting plasmon–exciton interaction in the intermediate coupling regime. *ACS Nano* **12**, 10393-10402 (2018).
36. Han, X. et al. Rabi splitting in a plasmonic nanocavity coupled to a WS_2 monolayer at room temperature. *ACS Photon*. **5**, 3970-3976 (2018).
37. Liu, X. et al. Nonlinear valley phonon scattering under the strong coupling regime. *Nat. Mater*. **20**, 1210-1215 (2021).
38. Deng, F. et al. Strong exciton–plasmon coupling in a WS_2 monolayer on Au film hybrid structures mediated by liquid Ga nanoparticles. *Laser Photonics Rev*. **14**, 1900420 (2020).
39. Zengin, G. et al. Approaching the strong coupling limit in single plasmonic nanorods interacting with J-aggregates. *Sci. Rep.* **3**, 1-8 (2013).
40. Zengin, G. et al. Realizing strong light-matter interactions between single-nanoparticle plasmons and molecular excitons at ambient conditions. *Phys. Rev. Lett*. **114**, 157401 (2015).
41. Wersall, M., Cuadra, J., Antosiewicz, T. J., Balci, S. & Shegai, T. J. N. l. Observation of mode splitting in photoluminescence of individual plasmonic nanoparticles strongly coupled to molecular excitons. *Nano Lett.* **17**, 551-558 (2017).
42. Liu, R. et al. Strong light-matter interactions in single open plasmonic nanocavities at the quantum optics limit. *Phys. Rev. Lett*. **118**, 237401 (2017).
43. Li, N. et al. Strong plasmon–exciton coupling in bimetallic nanorings and nanocuboids. *J. Mater. Chem. C* **8**, 7672-7678 (2020).
44. Schlather, A. E., Large, N., Urban, A. S., Nordlander, P. & Halas, N. J. Near-field mediated plexcitonic coupling and giant Rabi splitting in individual metallic dimers. *Nano Lett.* **13**, 3281-3286 (2013).
45. Roller, E.-M., Argyropoulos, C., Högele, A., Liedl, T. & Pilo-Pais, M. J. Plasmon–exciton coupling using DNA templates. *Nano Lett.* **16**, 5962-5966 (2016).
46. Chen, X. et al. Mode modification of plasmonic gap resonances induced by strong coupling with molecular excitons. **17**, 3246-3251 (2017).
47. Huang, J., Traverso, A. J., Yang, G. & Mikkelsen, M. H. Real-time tunable strong coupling: from individual nanocavities to metasurfaces. *Nano Lett.* **6**, 838-843 (2019).
48. Santhosh, K., Bitton, O., Chuntonov, L. & Haran, G. J. Vacuum Rabi splitting in a plasmonic cavity at the single quantum emitter limit. *Nat. Commun*. **7**, 11823 (2016).
49. Bitton, O. et al. Vacuum Rabi splitting of a dark plasmonic cavity mode revealed by fast electrons. *Nat. Commun*. **11**, 487 (2020).
50. Luo, Y. et al. Colloidal Assembly of Au–Quantum Dot–Au Sandwiched Nanostructures with Strong Plasmon–Exciton Coupling. *J. Phys. Chem.* **11**, 2449-2456 (2020).
51. Park, K.-D. et al. Tip-enhanced strong coupling spectroscopy, imaging, and control of a single quantum emitter. *Sci. Adv.* **5**, 5931 (2019).
52. Katzen, J. M. et al. Strong coupling of carbon quantum dots in plasmonic nanocavities. *ACS Appl. Mater. Interfaces* **12**, 19866-19873 (2020).
